# Plasminogen Deficiency Causes Reduced Corticospinal Axonal Plasticity and Functional Recovery after Stroke in Mice

**DOI:** 10.1371/journal.pone.0094505

**Published:** 2014-04-14

**Authors:** Zhongwu Liu, Yi Li, Jianyong Qian, Yisheng Cui, Michael Chopp

**Affiliations:** 1 Department of Neurology, Henry Ford Hospital, Detroit, Michigan, United States of America; 2 Department of Physics, Oakland University, Rochester, Michigan, United States of America; Hertie Institute for Clinical Brain Research, University of Tuebingen., Germany

## Abstract

Tissue plasminogen activator (tPA) has been implicated in neurite outgrowth and neurological recovery post stroke. tPA converts the zymogen plasminogen (Plg) into plasmin. In this study, using plasminogen knockout (Plg^-/-^) mice and their Plg-native littermates (Plg^+/+^), we investigated the role of Plg in axonal remodeling and neurological recovery after stroke. Plg^+/+^ and Plg^-/-^ mice (n = 10/group) were subjected to permanent intraluminal monofilament middle cerebral artery occlusion (MCAo). A foot-fault test and a single pellet reaching test were performed prior to and on day 3 after stroke, and weekly thereafter to monitor functional deficit and recovery. Biotinylated dextran amine (BDA) was injected into the left motor cortex to anterogradely label the corticospinal tract (CST). Animals were euthanized 4 weeks after stroke. Neurite outgrowth was also measured in primary cultured cortical neurons harvested from Plg^+/+^ and Plg^-/-^ embryos. In Plg^+/+^ mice, the motor functional deficiency after stroke progressively recovered with time. In contrast, recovery in Plg^-/-^ mice was significantly impaired compared to Plg^+/+^ mice (p<0.01). BDA-positive axonal density of the CST originating from the contralesional cortex in the denervated side of the cervical gray matter was significantly reduced in Plg^-/-^ mice compared with Plg^+/+^ mice (p<0.05). The behavioral outcome was highly correlated with the midline-crossing CST axonal density (R^2^>0.82, p<0.01). Plg^-/-^ neurons exhibited significantly reduced neurite outgrowth. Our data suggest that plasminogen-dependent proteolysis has a beneficial effect during neurological recovery after stroke, at least in part, by promoting axonal remodeling in the denervated spinal cord.

## Introduction

One of the most common impairments after stroke is hemiparesis of the contralateral body side to the affected cerebral hemisphere. As hemiparesis after stroke is a consequence of interruption of motor signals from the motor cortex to the spinal motoneurons, reestablishment of the corticospinal innervation provides a physical substrate for functional recovery. Our prior studies have demonstrated that axonal remodeling of the corticospinal tract (CST) contributes to neurological recovery after stroke in rodents [1]. In addition, bone marrow stromal cells (MSCs) significantly enhanced CST axonal outgrowth in the denervated spinal cord, and thereby improved motor functional recovery of the stroke-impaired forelimb [2]. Recent in vitro and in vivo data suggest that endogenous tPA mediates MSC induced neurite outgrowth and functional recovery after stroke [3]–[5]. Subacute (7 day post stroke) intranasal tPA delivery into the rodent brain also promoted CST axonal remodeling and behavioral outcome after stroke [6].

tPA was originally identified as a serine protease that catalyzes the conversion of the zymogen plasminogen (Plg) into the active plasmin [7]. In addition to its well established role in intravascular thrombolysis in the circulation system of the hepatic derived Plg, neuroendocrine tissue synthesized PA/Plg is widely distributed in the hippocampus, amygdala, hypothalamus, cerebellum, and cortex [8], [9], and is involved in axonal outgrowth and pathfinding [10], [11], synaptic plasticity [12], dendritic remodeling [13], and long term potentiation including learning and memory [7]. tPA has both proteolytic and non-proteolytic effects in the central nervous system (CNS). However, whether the tPA/plasmin system contributes to neurological recovery during the late phase after stroke, has not been explicitly investigated. To test whether the neurorestorative effects of tPA directly depend on the proteolytic action of tPA on plasminogen, we compared the behavioral outcome and CST axonal remodeling between Plg-deficient (Plg^-/-^) and Plg-native (Plg^+/+^) control mice subjected to middle cerebral artery occlusion (MCAo), and the status of neurite outgrowth primary cultured cortical neurons harvested from embryonic Plg^+/+^ and Plg^-/-^ mice.

## Materials and Methods

### Animal stroke model

Plg^-/-^ mice, B6.129P2-Plg^tm1Jld^/J [14], and wild-type (WT) mice, C57BL/6, purchased from Jackson Laboratory (Bar Harbor, ME) were mated to generate Plg heterozygous (Plg^+/–^) mice (F_1_ generation). The Plg^+/–^ mice were intercrossed and their F_2_ offspring genotyped by Southern blot analysis of tail-tip DNA. Male Plg^−/−^ mice and their corresponding Plg^+/+^ littermates at 8-10 weeks of age were subjected to permanent right intraluminal monofilament MCAo [15]. Plg^−/−^ mice exhibiting rectal prolapse before or during the experiments were excluded. Within the first week after surgery, five mice died out of the 25 subjected to MCAo (2 in Plg^+/+^ group and 3 in Plg^−/−^ group). All experiments were conducted in accordance with protocols approved by the Institutional Animal Care and Use Committee of Henry Ford Hospital (Permit Number: 1048). All surgery was performed under isoflurane anesthesia, and all efforts were made to minimize suffering.

### Behavioral measurements

The neurological functional deficits and recovery after stroke were monitored with a series of tests, i.e. foot-fault test [16] to assess the accuracy of left forepaw placement on a non-equidistant grid by the percentage of foot faults of the left forepaw to total steps, and single-pellet reaching test [17] to assess skilled reaching ability of the stroke-impaired left forepaw by success rate (%)  =  (number of pellets extracted/number of left forepaw attempts) x100. The tests were performed at 1 day prior to MCAo, and at 3, 7 days after stroke and weekly thereafter.

### Anterograde CST tracing

Fourteen days after MCAo, a unilateral craniotomy was performed over the left frontal motor cortex with a high speed drill. Ten % solution of biotinylated dextran amine (BDA, 10000 MW; Molecular Probes, Eugene, OR) in saline was injected through a finely drawn glass capillary into 4 points in the left frontal motor cortex of forelimb motor area (100 nl per injection site; stereotaxic coordinates: 0 and 0.5 mm rostral to the bregma, 1.5 and 2.0 mm lateral to the midline, 0.7 mm deep from the cortical surface) [18] to anterogradely label the CST axons originating from the pyramidal neurons in these areas.

### Tissue preparation and data analysis

Mice were perfused transcardialy with saline, followed by 4% paraformaldehyde at 28 days after MCAo (n = 10/group). The brain was cut into 7 equally spaced (1 mm) coronal blocks and embedded in paraffin, then sectioned for lesion volume measurement with hematoxylin and eosin staining. The cervical spinal cord segments of C4-7 were processed for vibratome traverse section (75 µm). Sections were incubated with primary antibody against biotin (Santa Cruz Biotechnology, Dallas, TX) for 3 days, and Cy-3 conjugated secondary antibody (Santa Cruz) overnight at 4°C. The sections were digitized with a laser-scanning confocal imaging system mounted onto a Zeiss microscope (Bio-Rad, Cambridge, MA). For each animal, the total length of midline-crossing axons in the denervated side of the ventral gray matter was measured on 10 z-axis image stacks with an ImageJ software plugin, NeuriteTracer.

### Primary culture of embryonic cortical neurons

Cortical neurons were harvested from pregnant female Plg^+/−^ mice at embryonic day 17–18. Briefly, under deep Ketamine anesthesia, embryos were removed and genotyped individually. The cerebral cortices of Plg^-/-^ and Plg^+/+^ embryos were dissociated in Ca^2+^ and Mg^2+^ free Hanks balance salt solution containing 0.125% trypsin digestion for 30 minutes. After filtered with cell strainers (BD Falcon REF 352350), the cells were seeded onto poly-D-lysine coated 6-well plates at a density of 5×10^4^ cells/well in Dulbecco's Modified Eagle's Medium (DMEM; Gibco, Grand Island, NY) containing 5% fetal bovine serum (FBS; Gibco). The cells were incubated at 37°C with 5% CO_2_ for 24 hours, and then transferred to Neurobasal medium (Gibco) containing 2% B-27 (Gibco) and 5% FBS.

The cells were fixed with 4% paraformaldehyde after 1 to 5 days in culture, respectively. The cells were incubated with monoclonal rabbit antibody against neuronal class III beta tubulin (Tuj1, 1∶1000; Covance, Princeton, NJ) and stained with Cy3-conjugated goat anti rabbit IgG (Jackson Immuno Research, West Grove, PA) to identify neurons. TuJ1-positive cells were digitized using a 20x objective (Zeiss) via the MicroComputer Imaging Device (MCID) analysis system (Imaging Research, St. Catharines, Ontario, Canada), and analyzed using MCID software for percentage of neurite positive neurons, branch number and neurite length on 100 neurons distributed in 9 random fields per well with three wells per group.

### Statistical analysis

The sample size (10 per group) and power of 80% were identified at the time of designing the experiments. The effect size was determined based on our many years of laboratory experience in stroke research. With 10 animals per group and 2 groups, we were able to detect an effect size of 1.325 using two sample t-test and considering an alpha = 0.05 and two-sided tests. All measurements were performed by experimenters blinded to each condition. Results are expressed as the mean ± SD. Significance of difference between animal groups was determined by one-way analysis of variance (ANOVA) followed by Tukey's post hoc test or unpaired Student's *t*-test. A value of P<0.05 was considered significant. To test the correlation between behavioral outcome and CST axonal remodeling, the correlation coefficients between the left forepaw motor performance and the axonal density in the denervated side of the cervical cord were calculated by Pearson's correlation coefficients.

## Results

### Lesion volume was not altered in Plg deficient mice after MCAo

**Figure 1:**
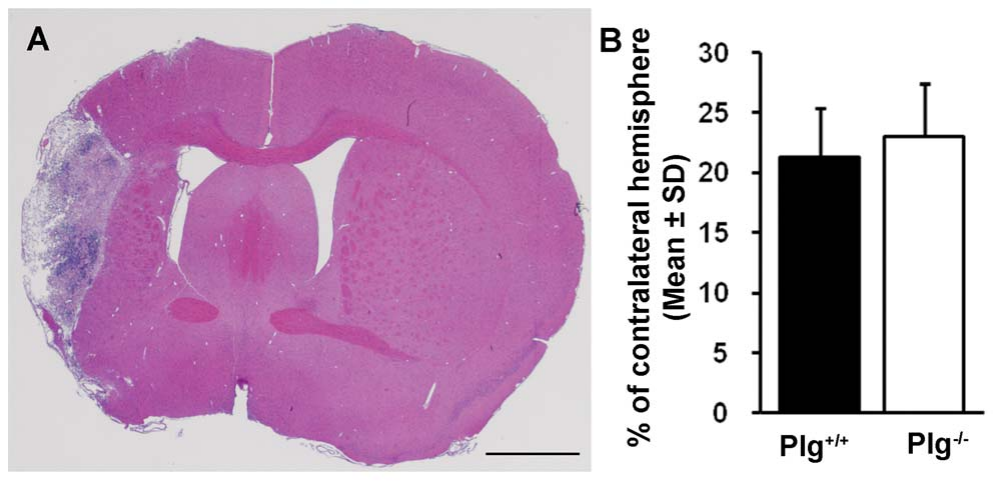
Ischemic lesion volume. A: A representative coronal section stained with hematoxylin and eosin shows ischemic infarct area. B: Quantitative data show there was no difference on the lesion volume between the Plg^+/+^ and Plg^-/-^ mice at 28 days after stroke (n = 10/group). Scale bar = 1 mm.

In both Plg^+/+^ and Plg^-/-^ mice subjected to permanent intraluminal monofilament MCAo, a large lesion area was observed in the cerebral cortex, corpus callosum, striatum, basal ganglia and thalamus at 28 days after stroke (Figure 1A). As quantitated as the percentage of the contralesional hemisphere, the infarction volumes were 21.3±4.1% (Range 16.1 to 27.4%) in Plg^+/+^ mice, and 23.0±4.4% (Range 14.7 to 28.6%) in Plg^-/-^ mice, respectively (B). There was no significant difference between groups.

### Motor functional recovery after stroke was reduced in Plg deficient mice

**Figure 2:**
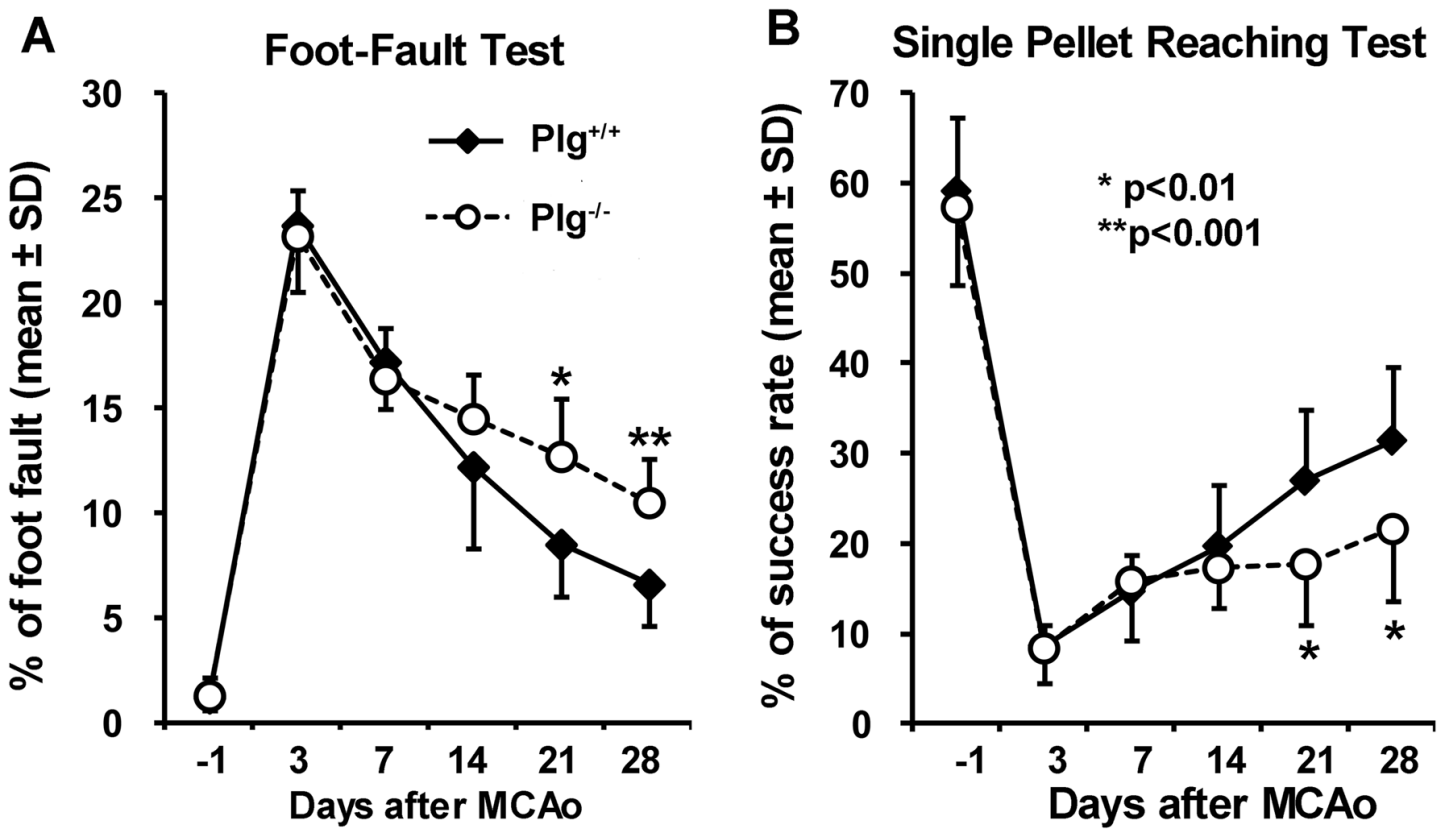
Behavioral outcome after MCAo. A: The foot-fault test measures the accuracy of forepaw placement on a non-equidistant grid as the percentage of foot-faults of the left forepaw to total steps. B: The single pellet reaching test measures the ability of skilled forepaw use. Animals were trained to use their left forepaw to extract food pellets through a vertical slot of the front wall. The number of the left forepaw extensions through the slot and the number of pellets extracted were counted. Performance was defined by the success rate as the percentage of pellet number extracted per left forepaw attempts. After stroke, significant behavioral deficits were evident in both tests. The Plg^+/+^ mice showed a progressive improvement with time, while the Plg^-/-^ mice exhibited a significant delayed recovery compared with the Plg^+/+^ mice (n = 10/group, *p<0.01, **p<0.001).

To measure the deficit and recovery of non-skilled and skilled motor performance of the stroke-impaired left forepaw, foot-fault test and single pellet reaching test were performed before and day 3 post stroke, and weekly thereafter. As shown in Figure 2, severe, however, comparable motor deficits were evident in both Plg^+/+^ and Plg^-/-^ mice after MCAo. The functional deficits gradually recovered with time; however, the recovery in Plg^-/-^ mice was significantly worse than in Plg^+/+^ mice post-stroke, assessed in both foot-fault test (A, p<0.01 at day 21 and p<0.001 at day 28) and single pellet reaching test (B, p<0.01 at day 21 and 28).

### Plg deficient mice exhibit reduced stroke-induced midline-crossing CST axonal growth into the denervated side of the cervical cord

**Figure 3:**
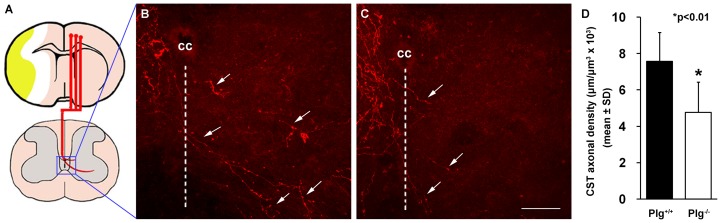
BDA-labeling of CST originating from the contralesional intact hemisphere. A: A schematic drawing shows BDA injection into the contralesional cerebral cortex and the location of pictures in B and C taken in the central area of the spinal gray matter. B and C: Representative confocal pictures from the Plg^+/+^ and Plg^-/-^ mice showing midline-crossing BDA-positive CST axons (arrows) sprouted into the denervated side of the ventral gray matter after stroke. D: Quantitative data showing that the length of BDA-labeled CST axons in the denervated side of the cervical cord was significantly decreased in Plg^-/-^ mice, compared with Plg^+/+^ mice (n = 10/group, *p<0.01). CC stands for central canal. Broken lines indicate the midline of the spinal cord. Scale bar = 50 µm.

Our prior studies demonstrated that unilateral cerebral stroke induces CST axonal remodeling in the spinal cord, namely, the CST axons originating from the contralesional cortex cross the midline of the spinal cord into the denervated side of the gray matter [19], and the midline-crossing CST axons contribute to behavioral recovery after stroke [20]. To characterize the anatomical basis of reduced motor recovery in mice lacking Plg, we injected BDA into the forelimb area of the contralesional cortex, to anterogradely label the CST axons (Figure 3A). In Plg^+/+^ mice subjected to MCAo, in the denervated side of the cervical cord, BDA-labeled CST axons crossed the midline of the spinal cord, and extended toward ventral horn (B). In contrast, BDA-labeled CST axons were rarely observed in the denervated spinal cord in Plg^-/-^ mice (C). Quantitative data showed that the density of BDA-labeled CST axons in the stroke-impaired side was significantly reduced in Plg^-/-^ mice (D, p<0.01).

### CST axonal remodeling highly correlates with behavioral outcome after stroke

**Figure 4:**
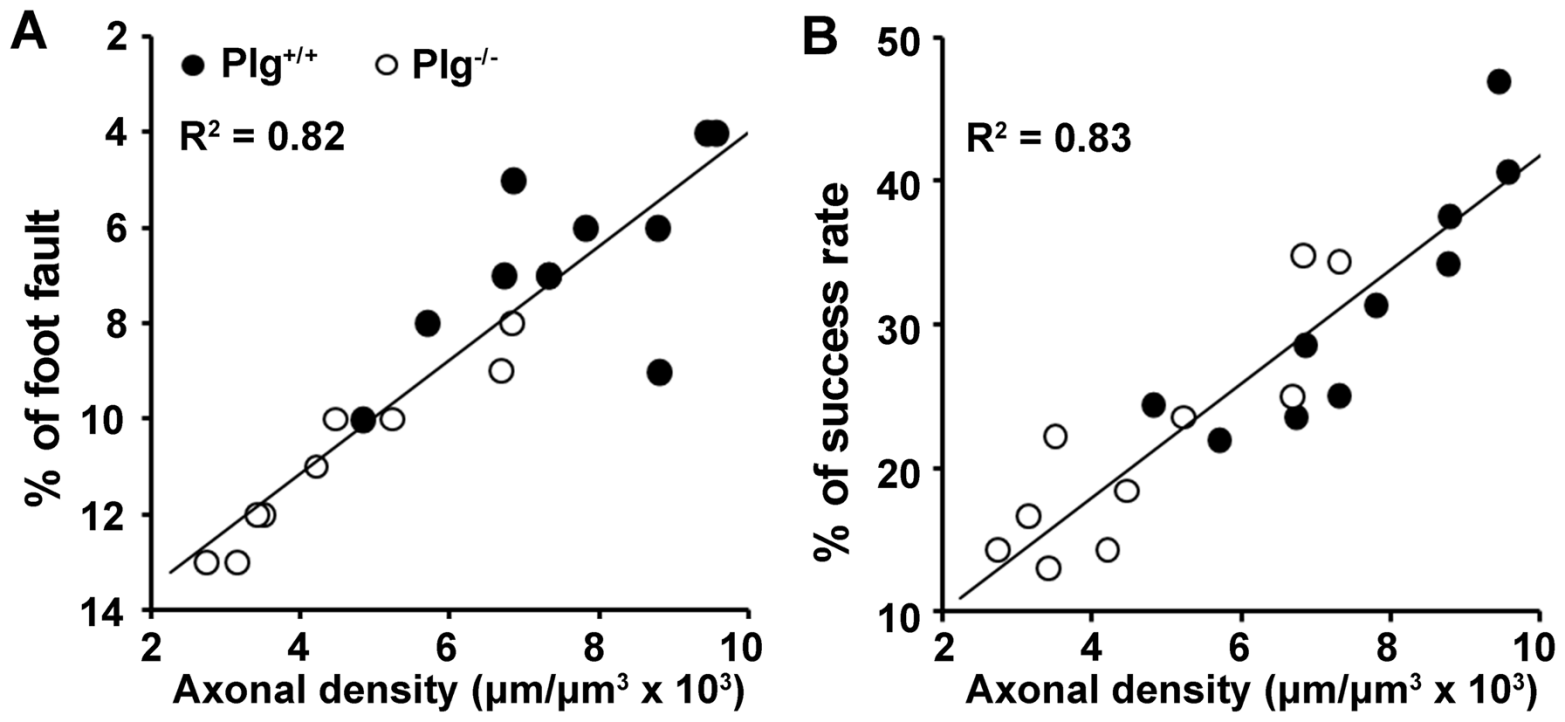
Data point graphs of correlations between axonal remodeling and behavioral recovery. The behavioral outcome assessed by both foot-fault (A) and single pellet reaching test (B) were highly correlated with the midline-crossing CST axonal density in the denervated side of the spinal cord (p<0.01).

To test whether contralesional CST axonal remodeling functionally contributes to neurological outcome after stroke, we examined the correlation of behavioral outcome with the midline-crossing CST axonal density in the denervated side of the cervical cord 28 days after MCAo. The data indicated that the motor performance of the stroke-impaired forelimb assessed with the foot-fault test and the single pellet reaching test were highly correlated with CST axons originating from the contralesional cortex (Figure 4A and B, R ^2^>0.82, p<0.01).

### Plg deficient cultured cortical neurons exhibit reduced neurite outgrowth

**Figure 5:**
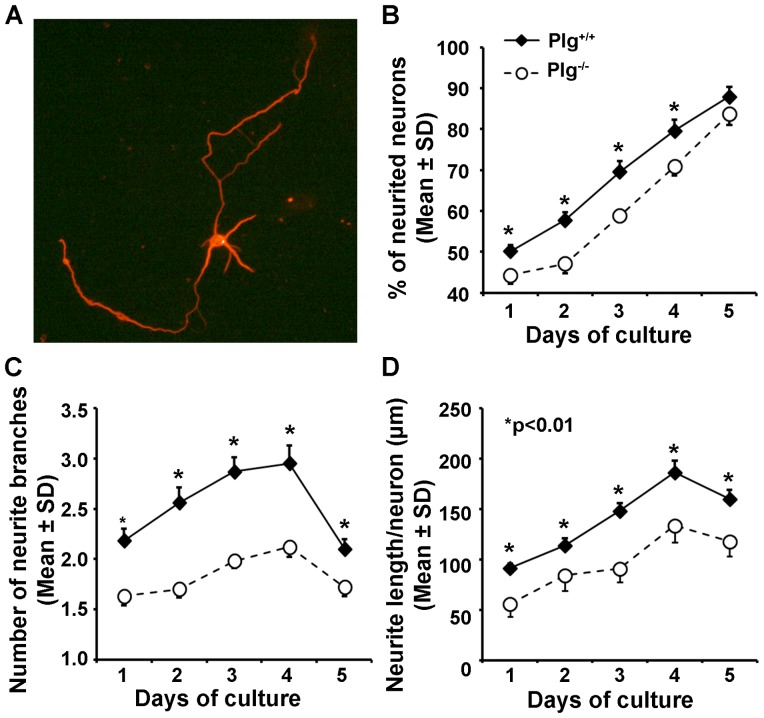
Neurite outgrowth in primary cultured cortical neurons harvested from Plg^+/+^ and Plg^-/-^ embryos. A representative image shows the neurons identified with immunofluorescent staining for beta tubulin (Tuj1, A). Compared to Plg^+/+^ neurons, the Plg^-/-^ neurons exhibited a significantly reduced neurite outgrowth measured in the percentage of neurite positive neurons (B), number of neurite (C) and neurite length (D, n = 100, *p<0.01).

To verify whether Plg deficiency alters the ability of neurite outgrowth, we compared primary cultured cortical neurons harvested from both Plg^+/+^ and Plg^-/-^ embryos (Figure 5). The neurons were identified with fluorescent immunostaining for neuronal Tuj1 (A). Throughout the culture period of 5 days, we found that the percentage of neurite positive neurons during day 1 to day 4 (B), and the number of neurite branches (C) and total length of neurites per neuron (D) were significantly reduced in the Plg^-/-^ neurons compared with Plg^+/+^ neurons (p<0.01).

## Discussion

In this study, to directly examine the importance of plasminogen in stroke onset and recovery, we investigated the differences of behavioral outcome and contralesional axonal remodeling of the CST in young adult Plg^-/-^ and genetically matched Plg^+/+^ mice subjected to MCAo, and neurite outgrowth in cortical neurons harvested from both Plg^+/+^ and Plg^-/-^ embryos. In mice lacking Plg, there are no obvious phenotypic abnormalities observed in the majority of animals up to 21 weeks of the age, except for inflammatory rectal lesions and rectal prolapse found in some animals [14]. Our data indicate that mice lacking Plg showed reduced neurological recovery and corticospinal motor axonal outgrowth in the denervated spinal gray matter. Additionally, primary cultured cortical neurons harvested from the Plg^-/-^ mice showed decreased neurite outgrowth compared to cortical neurons from Plg^+/+^ mice.

The PA/Plg system, in addition to fibrinolyis, plays a key role in the development of the nervous system [21] and possibly in mediating neuronal plasticity post stroke. In the current study, in mice subjected to permanent intraluminal MCAo, the ischemic lesion volumes were comparable between Plg^+/+^ and Plg^-/-^ mice at day 28 post stroke. In an early study of mice subjected to ligation of distal MCA, the focal cerebral infarct size at 24 hours after MCAo was significantly larger in mice with Plg deficiency than WT mice [22]. Although the reasons for the apparent discrepancy with the earlier observations on infarct volume need to be further investigated, they may be related to differences in MCAo surgical procedures, and the 24 hour time point of sacrifice in the distal MCA ligation study, which may have been too early to identify a mature ischemic infarct. Our consistent observations on the comparable severity of behavioral deficit at the early phase, i.e., day 3 after stroke, suggests that Plg deficiency may not, at least in the permanent intraluminal MCAo model, significantly affect the infarct volume. In addition, tPA induces neuronal excitotoxicity through binding to and cleavage of the NR1 subunit of the N-methyl-D-aspartate receptors [23]; however, the neurotoxic role of tPA in ischemic tissues remains uncertain. An early study showed that transient ischemia/reperfusion in SV129 background tPA-deficient mice exhibited approximately 50% smaller cerebral infarcts than in C57BL/6 wild-type mice [24]. In contrast, a similar study showed that the infarct volume in tPA^-/-^ mice was larger than background matched tPA^+/+^ mice, however, infarct volume was much smaller than in C57BL/6 mice [25]. Our results of comparable infarct volume in background matched Plg^-/-^ and Plg^+/+^ mice suggest that the proteolytic function of plasmin may not be directly involved in the potential neurotoxicity of tPA during the evolution of the ischemic infarct.

Patients with Plg deficiency exhibit symmetric internal hydrocephalus with a Dandy-Walker malformation, hypoplasia of the cerebellum, and a hypoplastic corpus callosum [26], indicating an important role of the Plg activation system in neuronal development. In both behavioral tests employed to estimate the neurological outcome of the left forepaw in mice subjected to right MCAo, the mice need to voluntarily control the paw movement. In the foot-fault test, when mice walk on the non-equidistant grids, each step requires adjustment in stride length and distribution of body weight, to place the limb appropriately on the rung and then to grasp it [27]. For the skilled reaching task, mice advance the forelimb aimed to the pellet, pronate the paw on it, grasp it, extract it and release the food into the mouth [17]. Our behavioral data showing no differences in motor performance between Plg^+/+^ and Plg^-/-^ mice before MCAo are in agreement with the observation that Plg deficiency does not alter neuromotor ability, motor coordination, locomotor activity, reaction to gravitational positioning, integration of motor and vestibular systems during postnatal development [21]. The observations of comparable infarct volume and severity of behavioral deficit at day 3 after stroke between Plg^+/+^ and Plg^-/-^ mice suggest the delayed recovery in Plg deficient mice is not attributed to differences in acute injury. Furthermore, the behavioral outcome was highly correlated with CST axonal remodeling in the denervated side of the spinal cord after stroke. Therefore, we suggest that the reduced behavioral recovery in Plg^-/-^ mice during the late phase (3 to 4 weeks) after stroke may be attributed to reduced neurological plasticity.

Unilateral stroke affects both sides of the brain. In neuroimaging studies of stroke patients, co-activation appears in bilateral motor areas when moving the affected limb [28]. Furthermore, the contralesional motor system may contribute to compensatory recovery of the affected forelimb [29]. Growth factors do not further increase axonal sprouting in the injured hemisphere, but promote lesion-remote plasticity of the contralesional pyramidal tract [30]. To investigate the neuroanatomical basis of the reduced motor functional recovery in Plg^-/-^ mice, we traced the CST axons originating from the contralesional forelimb motor area with intracortical injection of anterograde neuronal tracer, BDA. Our previous study demonstrated that stroke induced interhemispheric axonal remodeling in the spinal cord [19]. The present data showed in the stroke-impaired side of the spinal gray matter, that midline-crossing CST axonal outgrowth was significantly reduced in Plg^-/-^ mice compared to their genetic background matched Plg^+/+^ mice. Interestingly, Plg activation is increased at the crush site of the sciatic nerve accompanying peripheral nerve regeneration [31], and Plg^-/-^ mice show delayed functional recovery after sciatic nerve crush [32], suggesting Plg may contribute to axonal regeneration in a common way in both the CNS and peripheral nervous system. In the present study, we primarily focused on the axonal remodeling of the direct motor pathway, the CST. However, the rubrospinal tract participates in the coordination of movements across joints, such as skilled forelimb movements [33], locomotion [34] and motor responses to pain [35], and possesses very similar branching patterns with the CST in the spinal cord [36]. The cortico-rubrospinal pathway appears to be a backup to the CST to enhance the behavioral recovery after CST lesion [37]. We and others have demonstrated that axonal plasticity of the corticorubral tract [38]–[40] and other spinal descending pathways [41] contribute to functional recovery after stroke in rodents. Further investigations on these alternate pathways to reveal the detailed anatomical substrates for tPA/Plg mediated axonal remodeling and neurological recovery after stroke are warranted.

In primary cultured embryonic cortical neurons, we found that Plg deficiency significantly reduced neuritogenesis, and neurite sprouting and outgrowth. tPA is primarily produced by neurons and microglia, whereas plasminogen is exclusively expressed by neurons [42]. Previous studies suggested that non-proteolytic effects of tPA derived from microglia may indirectly affect hippocampal mossy fiber pathfinding and outgrowth [43], while the neuron derived tPA/plasmin proteolytic system facilitates continued neurite extension via degradation of the extracellular matrix proteoglycans and cell surface components [44]. In addition, it has been demonstrated that cleavage of the precursor brain-derived neurotrophic factor (BDNF) into the mature BDNF by the extracellular protease plasmin is essential for long term hippocampal plasticity [45]. The tPA–plasmin cascade has also been implicated in cleavage of the precursor nerve growth factor (NGF) to mature NGF [46], [47]. Furthermore, Plg gene expression is regulated by NGF [48], and is required in NGF induced neuritogenesis [49] and neurite outgrowth [48]. Blockade of the proteolytic activity of plasmin delayed NGF-dependent neuritogenesis and neurite outgrowth [49]. Therefore, we suggest that the reduced neurological recovery after stroke in Plg deficient mice may be attributed to direct participation of plasmin proteolysis in neuritogenesis and neurite outgrowth. However, in the present study, we did not investigate the effects of tPA/Plg on dendrites, which may be regulated by different mechanisms during development and plasticity [50], [51]. Further studies to specifically investigate the distinct effects of tPA/Plg on axonal and dendritic outgrowth and plasticity are warranted.

## Conclusions

Taken together, the present observations of reduced behavioral outcome and axonal remodeling in Plg deficient mice demonstrate that the endogenous Plg-dependent proteolysis is an important element involved in neurological recovery after stroke, suggesting that, in addition to being used as a thrombolytic agent in the circulation system, tPA/plasmin in the CNS parenchyma is neurorestorative, and provides therapeutic benefit by enhancing neuronal remodeling during the convalescence after stroke.
